# Improving the prediction of mRNA extremities in the parasitic protozoan *Leishmania*

**DOI:** 10.1186/1471-2105-9-158

**Published:** 2008-03-20

**Authors:** Martin Smith, Mathieu Blanchette, Barbara Papadopoulou

**Affiliations:** 1 Research Centre in Infectious Diseases, CHUL Research Centre, 2705 Laurier Blvd., Quebec, QC G1V 4G2, Canada; 2 McGill Center for Bioinformatics, 3775 University St., Montreal, QC H3A 2B4, Canada

## Abstract

**Background:**

*Leishmania *and other members of the *Trypanosomatidae *family diverged early on in eukaryotic evolution and consequently display unique cellular properties. Their apparent lack of transcriptional regulation is compensated by complex post-transcriptional control mechanisms, including the processing of polycistronic transcripts by means of coupled *trans*-splicing and polyadenylation. *Trans*-splicing signals are often U-rich polypyrimidine (poly(Y)) tracts, which precede AG splice acceptor sites. However, as opposed to higher eukaryotes there is no consensus polyadenylation signal in trypanosomatid mRNAs.

**Results:**

We refined a previously reported method to target 5' splice junctions by incorporating the pyrimidine content of query sequences into a scoring function. We also investigated a novel approach for predicting polyadenylation (poly(A)) sites *in-silico*, by comparing query sequences to polyadenylated expressed sequence tags (ESTs) using position-specific scanning matrices (PSSMs). An additional analysis of the distribution of putative splice junction to poly(A) distances helped to increase prediction rates by limiting the scanning range. These methods were able to simplify splice junction prediction without loss of precision and to increase polyadenylation site prediction from 22% to 47% within 100 nucleotides.

**Conclusion:**

We propose a simplified *trans*-splicing prediction tool and a novel poly(A) prediction tool based on comparative sequence analysis. We discuss the impact of certain regions surrounding the poly(A) sites on prediction rates and contemplate correlating biological mechanisms. This work aims to sharpen the identification of potentially functional untranslated regions (UTRs) in a large-scale, comparative genomics framework.

## Background

*Leishmania *is a unicellular eukaryote that belongs to the *Trypanosomatidae *family; a strictly parasitic order of *kinetoplastida*. *Leishmania *is the causative agent of leishmaniases, vector-borne parasitic diseases with a large spectrum of clinical manifestations in humans ranging from self-resolving skin lesions to life-threatening visceral diseases [1]. Leishmaniasis is endemic in 88 countries mainly in tropical and subtropical regions with an estimated 12 million people presently infected worldwide and at least 350 million people being at risk of infection [2].

Trypanosomatid protozoan parasites have diverged early on in eukaryotic evolution [3]. Their evolutionary closeness to bacterial ancestors is delineated by intrinsic cellular characteristics such as tandem arranged genes [4], polycistronic transcription [5,6], mitochondrial RNA editing [7], lack of transcriptional control [8], infrequent introns [9], and *trans*-splicing [10]. The latter consists of the 5' cleavage of polycistronic RNA precursors into individual mRNA transcripts by addition of an exogenous 39 to 41 base long capped RNA fragment, namely the splice leader (SL) or mini-exon, provided by a highly abundant SL-RNA [11], yet similar processes have also been discovered in nematodes and even in mammals [12,13]. This process is somewhat similar to *cis*-splicing in other organisms, as RNA is cleaved at an AG dinucleotide downstream of a polypyrimidine stretch.

In addition to co-transcriptional *trans*-splicing, polyadenylation of the upstream transcript is also required in order to generate monocistronic mRNAs in these organisms. Trypanosomatid protozoa are believed to lack a conserved polyadenylation (poly(A)) signal, in contrast to other eukaryotes who generally require a cytoplasmic polyadenylation motif for successful polyadenylation [14]. Several studies support that polyadenylation is mechanistically coupled to *trans*-splicing and that it depends upon the presence of polypyrimidine tracts [15-19], thus leading to the belief that the spliceosome complex interacts with the polyadenylation machinery in trypanosomatids. It has also been reported that distant pyrimidine tracts may be responsible for polyadenylated positions further away from the downstream 5' splice site in trypanosomes [17,20]. These analyses also convey the non-specific nature of poly(A) site selection in trypanosomatids, as polyadenylation seems to occur in a given region rather than at a specific position.

The apparent heterogeneity of kinetoplastid mRNA polyadenylation and its dependence on successful *trans*-splicing make 3'-untranslated region (3'UTR) length predictions troublesome. Currently, there exists a 3'UTR prediction method for *Trypanosoma brucei *derived from the statistical analysis of mRNA transcript extremity lengths from expressed sequence tag (EST) data [20]. The prediction is essentially obtained by selecting the position located at an empirical distance (100 bases) upstream of the polypyrimidine tract closest to the open reading frame (ORF). The authors claim a 38% prediction rate within a 73-nucleotide window. These metrics are somewhat inappropriate for predictions in the *Leishmania *genus since the species flaunt larger intergenic (IR) sequences, higher average UTR lengths, and less stringent splice acceptors [4,21].

In addition to the statistical analysis of transcript length distributions for 3'UTRs, 5'UTR prediction has been submitted to supplementary investigation [22-24]. Prediction algorithms that essentially focus on selecting the first AG dinucleotide after the longest polypyrimidine stretch can reportedly identify 62% of valid splice junctions in trypanosomes and 51% in *Leishmania *[20,23]. For *Leishmania*, it has been shown that by fragmenting the non-coding sequence upstream of a start codon at every occurrence of AG, the AG following the longest fragment corresponds to a valid splice junction in 60% of the cases. When combining this method with a linear discriminant analysis of dinucleotide composition, the later method can obtain a prediction accuracy as high as 92% on selected high-scoring sequences [23].

Considering that regulation of gene expression in kinetoplastids occurs mostly at the post-transcriptional level, it has become apparent that UTRs bear essential regulatory tags [8,25-29]. From the standpoint of computational motif discovery, it is imperative to discriminate between functional and non-functional sequences in order to successfully identify novel conserved regulatory regions. This premise is most important when dealing with non-coding RNA as it is exposed to less stringent evolutionary pressure than open reading frames [30]. It can be expected that limiting sequence and structure motif searches to legitimate mRNA UTRs will generate more informative results while reducing the inherent computational cost of search algorithms.

This paper aims to further improve the *in-silico *prediction of mRNA extremities in kinetoplastid organisms. We polish *trans*-splicing prediction in *Leishmania *by incorporating the pyrimidine content of intergenic regions into a previously developed scoring function, and propose a polyadenylation prediction method based on the global nucleotide composition observed in published expressed sequence tag (EST) data. The selection of different genomic regions surrounding the poly(A) site and their impact on prediction rates has validated the impact of adenosines and downstream polypyrimidines on trypanosomatid polyadenylation.

## Results

### Considering pyrimidine content increases splice-junction prediction accuracy

Previously, the best method to predict splice acceptor sites in trypanosomatids combined statistical analysis of dinucleotide composition with inter-AG fragment length assessment [23]. We simplified the procedure by discarding the statistical discrimination of inter-AG fragments based on dinucleotide composition, thus only considering the inter-AG fragment size for predictions. This approach was compared to two pyrimidine-bias scoring functions that rate inter-AG segments in proportion to their pyrimidine content in addition to their size (see Methods). Both functions are such that inter-AG fragments displaying lower than average pyrimidine content are proportionately penalised whereas those with higher than average content are rewarded.

Each scoring model's relative sensitivity with respect to a set of 214 known splice junctions is compared in Table 1. It appears that models that consider pyrimidine concentration can predict more valid splice junctions than those using the inter-AG length metric alone. The proportion of valid predictions is notably higher (+7%) when allowing a 25-nucleotide margin of error. This is not surprising as it is common for more than one AG dinucleotide to be in close range of each other near splice acceptor sites (data not shown). The pyrimidine-bias scoring functions were compared to the full inter-AG and linear discriminant analysis using the same reported search space (400 nt upstream of the splice junction). Both methods offer similar predictions although the pyrimidine bias functions display slightly higher rates (+2%). The linear pyrimidine scoring function was chosen for subsequent analyses given its accuracy and simplicity.

**Table 1:** Splice junction prediction sensitivities of three different scoring models.

**Scoring Function**	**Exact**	**< 25 nt**
Longest Inter-AG Length	49.5	58.9
Linear *Y*-bias	53.7	65.9
Polynomial *Y*-bias	53.3	64.0

Inter-AG Length + LDA*	58.6	72.1
Linear *Y*-bias**	58.9	74.3
Polynomial *Y*-bias**	60.7	74.3

* Interpreted from [23]

** Using same search space as Inter-AG + Linear Discriminant Analysis

Values are the ratio (%) of correct predictions among the 214 sequences in the test set, for exact predictions and predictions within 25 bases of the sequenced 5' splice junction. The upper half displays values obtained using a search space of 700 whereas the lower half displays predictions using the same query size as the linear discriminant analysis.

### Nucleotide composition shifts surrounding the genomic poly(A) site

Of the 12,052 *Leishmania *EST sequences in GenBank, 81% correspond to *L. infantum *and 19% to *L. major *cDNA. We filtered the data to collect sequences harbouring significant poly-A or poly-T stretches near their extremities in order to search for polyadenylation signals. Only 850 sequences (7% of initial data) satisfied our search constraints (see Methods) of which a mere 218 (1.8%) were successfully mapped to genomic intergenic regions of *L. infantum *(the accession numbers for the 218 ESTs can be viewed in Additional File 1). The *L. infantum *EST data contains several flagrantly erroneous and repeated sequences. Comparing the pair-wise identity of mapped ESTs revealed 4 pairs of highly similar sequences which, once aligned, proved to be the only example of alternatively polyadenylated sequence in our data (GenBank accession IDs: CV669949.1, CV670417.1, CV668181.1, CV665773.1, CV670284.1, CV668879.1, CV667130.1, CV661593.1).

**Figure 1:**
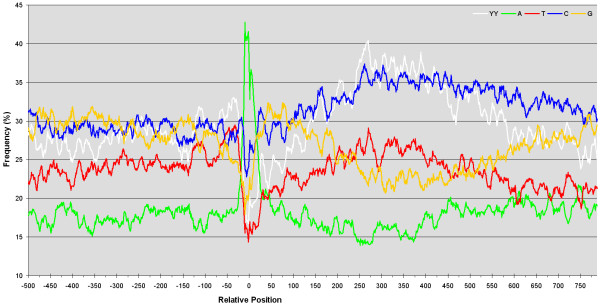
**Nucleotide and pyrimidine dinucleotide frequencies surrounding the mapped polyadenylation site of 218 expressed sequence tags from *Leishmania infantum***. Frequencies are averaged over an 11 nucleotide sliding window in order to smooth out the graph. Negative positions are 5' of the poly(A) site and positive positions are towards the downstream gene. Pyrimidine dinucleotides are considered to be any occurrence of consecutive C or T residues.

The position-specific nucleotide frequencies of genomic regions aligned and centered at the mapped poly(A) position reveals prominent trends in global sequence composition (Figure 1). Adenosine residues are bountiful near the poly(A) site and an elevated concentration of pyrimidines is perceptible 300 to 600 bases downstream of it. Interestingly, thymine bases are almost twice as abundant around 50 bases upstream of the poly(A) site and their higher overall concentration is synonymous with that of pyrimidine dinucleotides. Not only are adenosine and pyrimidine nucleotides more abundant in polyadenylated regions, they also fluctuate more than that of randomly selected genomic sequences (Table 2). When comparing the standard deviations of residues near poly(A) sites, pyrimidine dinucleotides (YY) have a higher standard deviation than their individual nucleotides alone. It is noteworthy to mention that the nucleotide frequencies tend to resemble that of the random control when extended further away from the poly(A) position.

**Table 2:** Global statistics of genomic sequences in *Leishmania infantum*.

	**Genomic Poly(A)**					**Random Genomic**				
	**A**	**T**	**C**	**G**	**YY**	**A**	**T**	**C**	**G**	**YY**
**Average**	20.3	25.6	34.1	29.3	29.1	20.1	20.2	29.7	30.0	24.1
**Median**	20.8	25.7	34.0	28.6	29.6	20.0	20.4	29.7	30.0	24.2
**Standard Deviation**	3.8	2.7	2.6	2.8	4.0	1.0	1.0	1.0	1.0	1.1

Values are derived from 218 sequences of 1601 nucleotides and represent the canonical DNA bases in addition to pyrimidine dinucleotides (YY). The genomic poly(A) sequences encompass 800 nucleotides surrounding the mapped poly(A) site. As a control, 218 genomic regions were selected at random via an *ad-hoc *JAVA script.

Capturing such blatant genomic signals in addition to more discrete parameters, like progressive shifts in nucleotide and dinucleotide compositions, could be an effective means of identifying poly(A) sites in unresolved sequences. Such a comparative approach is appealing since conserved sequence motifs surrounding poly(A) sites in trypanosomatid species are not as common as in higher eukaryotes. Using motif detection programs such as MEME [31] did not yield conclusive results (data not shown). Indeed, the intergenic regions of *Leishmania *parasites are riddled with low-complexity regions (i.e., short consecutive repeats of 1–3 nucleotides) that can bias the scoring metrics of such programs. To surmount this shortcoming, we investigated over-represented motifs in the regions directly surrounding genomic poly(A) sites in *Leishmania *using the word enumeration program YMF [32] in combination with FindExplanator [33]. Hexamers that are over-represented in the regions directly flanking known genomic poly(A) sites were compared to those found in more distant regions (see Additional File 2 for details). The highest-ranking motifs are present in only a fraction of all known poly(A) sites and appear to be randomly distributed within their vicinity (data not shown).

### Poly(A) sites can be predicted using scanning matrices

We converted the genomic alignment into a position specific scoring matrix (PSSM) that can subsequently be used to scan non-coding sequences. The PSSM is aligned to every position within the intergenic sequence and emits a bit-score for each position (see Methods). The higher the score, the closer the current position in the intergenic sequence resembles the global composition of a polyadenylated region. We present the depicted prediction rates of a given PSSM as a measure of its sensitivity, or ability to detect valid poly(A) sites. Since the biological data is limited, sensitivity was determined using tenfold cross-validation (see Methods) which allows for unbiased testing, as the testing data is excluded from the training data [34]. The position displaying the highest PSSM score is retained as the poly(A) candidate.

**Figure 2:**
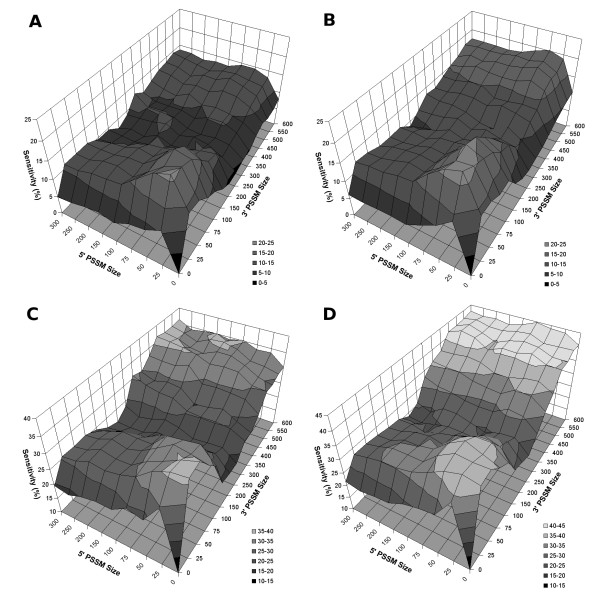
**Surface plots of poly(A) prediction sensitivities as a function of various PSSMs**. (**A**) Predictions within 10 bases of sequenced poly(A) site when scanning the entire intergenic region (IR) and (**B**) when limiting scanning to 100 bases upstream of the predicted splice junction. Predictions within 100 bases when scanning the whole IR (**C**) and constrained by 1000 bases of the predicted splice junction (**D**). Sensitivities are presented as the average of 15 runs of ten fold cross-validation for each PSSM. The 5' and 3' PSSM size axes correspond to the region upstream and downstream of the genomic alignment of mapped poly(A) sites, respectively. In order to amplify the resolution of regions directly surrounding the poly(A) sites, the scale for 5' and 3' matrix sizes inferior to 100 is decreased from 50 to 25.

Given that the molecular mechanisms of kinetoplastid polyadenylation have yet to be completely demystified, we tested multiple PSSM lengths in order to elucidate which regions surrounding the cleavage site have an effect on polyadenylation. Matrix sizes were limited to regions where a meaningful base composition pattern was observed. The most precise predictions are obtained with small PSSMs encompassing the adenosine rich region directly surrounding the aligned poly(A) sites (Figure 2A). Using a prediction tolerance of ≤10 nucleotides, a PSSM of 25 bases upstream and 25 bases downstream of the poly(A) site (25A25) shows the highest sensitivity (21% average after 15 runs of 10-fold cross-validation), with a standard deviation of 1.1%. At lower resolutions, the same small PSSMs still display the best predictions, however longer matrices such as the 300 upstream and 600 downstream PSSM (300A600) offer similar sensitivities (Figure 2C). Overall, the surface plots show that the regions adjacent to the poly(A) site offer the highest close-range predictions when scanning entire intergenic regions, although larger matrices also display competitive detection rates provided that the margin of error is relaxed.

### Limiting PSSM scanning range increases poly(A) site prediction rates

**Figure 3:**
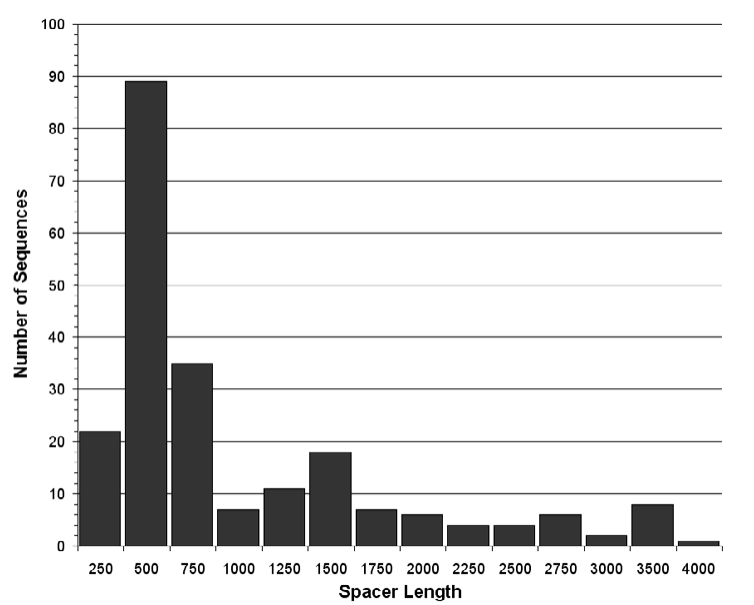
**Distribution of spacer sequences**. Distribution of the distance between the mapped polyadenylation site and the putative splice junction (spacer length) of 218 intergenic regions from *Leishmania infantum*. *Trans*-splicing positions were estimated using the linear pyrimidine bias function described in Methods.

In order to maximize the sensitivity of poly(A) site targeting, we tested the impact of bounding the PSSM search space within a given confidence interval. To do so, the aforementioned refined splice-junction prediction method was applied to the intergenic sequences derived from the polyadenylated ESTs in order to obtain an approximation of the distances between both cleavage sites. The distribution of the putative intergenic spacers shows that 83% of the spacer sequences are shorter than 1500 bases (Figure 3), with a median value of 498.

**Figure 4:**
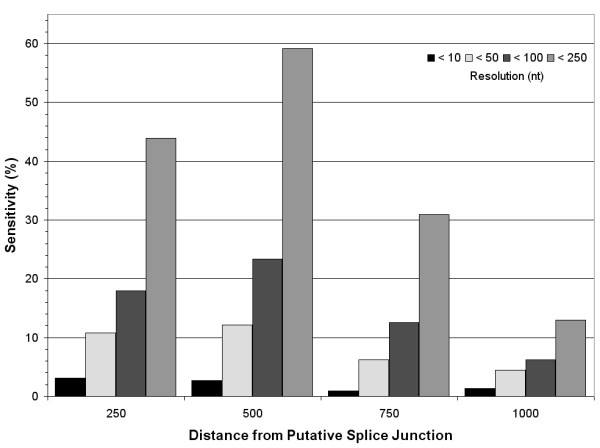
**Prediction sensistivities using fixed distances**. Sensitivity of poly(A) site predictions using fixed distances from the putative splice junction of 218 intergenic regions mapped from polyadenylated ESTs in *Leishmania infantum*. The resolution corresponds to the distance allowed between the true poly(A) site and the predicted poly(A) site. Standard deviations are denoted as the bars above each column.

Based on these observations, it is clear that distance is an important factor to incorporate into an mRNA extremity prediction algorithm. We tested the effect of predicting 3'UTR extremities using splice junction prediction combined to a fixed distance as the prime metric. The highest prediction accuracies using this approach are obtained by selecting the median value of spacer sequence sizes as a scanning limit (Figure 4). When allowing predictions to be within 100 bases of the valid poly(A) site, this tactic predicts 22% of valid splice sites. At this resolution, scanning the entire IR with PSSMs yields a 36% detection rate, more than double the distance-only value.

**Figure 5:**
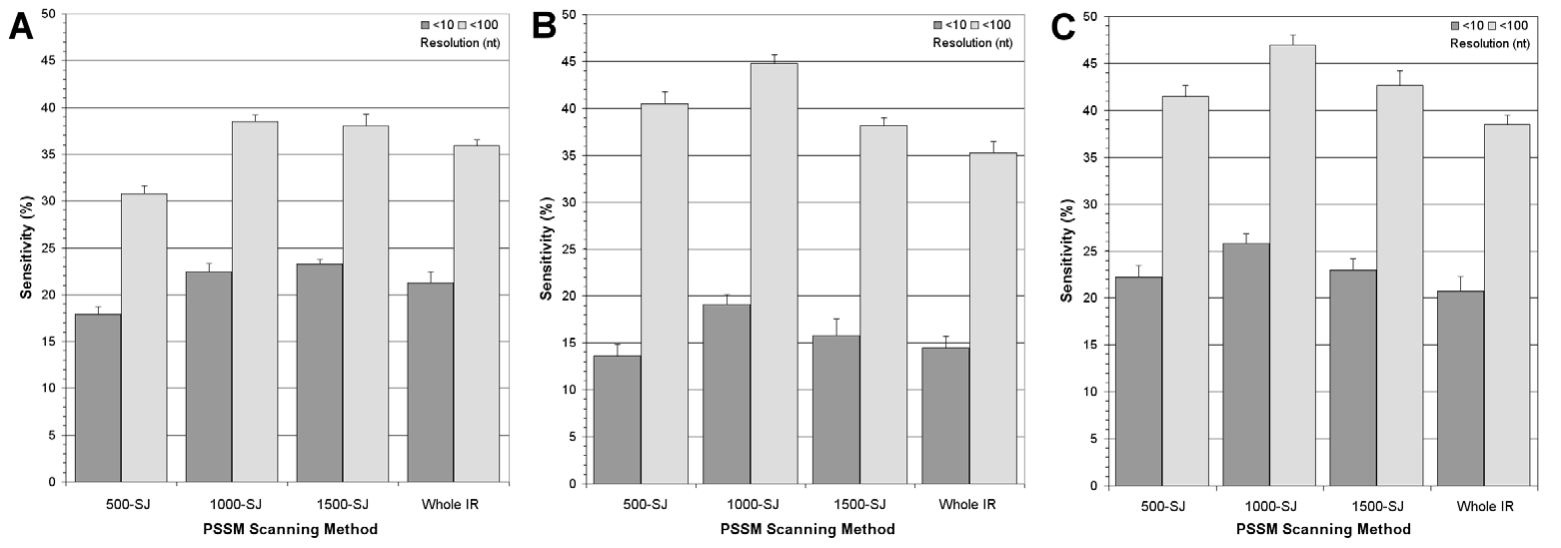
**Comparison of poly(A) prediction sensitivities for chosen PSSMs using different scanning approaches**. The mean sensitivities after 15 runs of tenfold cross-validation are presented for whole intergenic region scanning and for 3 limited scanning ranges (from the putative splice-junction to 500, 1000, and 1500 positions upstream. (**A**) Mean sensitivities using a PSSM size of 25A25 (25 bases upstream and downstream of the mapped poly(A) position). (**B**) Mean sensitivities using a PSSM size of 75A600. (**C**) Mean sensitivities using a combination of both PSSMs (see Results for details).

We subsequently scrutinized the prediction rates for all PSSMs using various scanning distance limitations, a handful of which are compared amongst themselves (Figure 5). The impact of limiting the scanning distance directly upstream of the putative splice-junction site produces a notable increase in sensitivity for most PSSMs. The overall highest sensitivities are obtained by limiting the scanning distance to within 1000 bases of the SJ. This is most notable for the longer matrices, some of which gained over 5% sensitivity within the 10-nucleotide range (Figure 2B), thus competing with the shorter matrices for the best prediction rate. At the 100-nucleotide range, limiting the scanning distance to within 1000 bases increased the sensitivity from 36% to almost 45% (Figure 2D). Curiously, matrices encoding the pyrimidine rich regions offer the highest sensitivities at this resolution whereas very small ones containing the A-rich region perform best within a 10 nucleotides error margin. When loosening the predictive resolution to within 250 bases, certain PSSMs (most notably 30A600 and 300A600) can identify slightly more than 60% of the mapped poly(A) sites (see Additional File 3 for all sensitivities).

**Figure 6:**
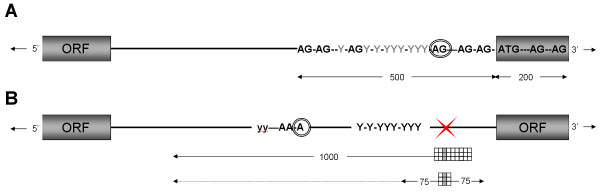
**Summary of PRED-A-TERM program**. (**A**) The putative *trans*-splicing site is first predicted by fragmenting 700 nucleotides at each occurrence of AG. The longest, pyrimidine rich inter-AG fragment is selected as the sequence upstream of the splicing site. (**B**) A large scanning matrix subsequently skims through 1000 nucleotides upstream of this position, identifying pyrimidine rich areas with adenosines upstream of them. For each large matrix position, a smaller matrix scans 75 positions in both directions from the larger matrix's hypothetical poly(A) site in order to pinpoint adenosine residues contrasted to pyrimidines. The position of the smaller matrix displaying the highest sum of bit-scores is retained as the putative poly(A) site.

We tested the effect of combining the high-resolution accuracy of the 25A25 matrix with the low-resolution accuracy of a larger matrix on prediction sensitivity. Two algorithms were tested. The first involves an initial scan with the large matrix, where the highest scoring position and its surrounding sequence are then re-scanned with the smaller matrix. The highest score from this second scan is reported as the presumed poly(A) site. Similarly, the second algorithm combines the scores of both PSSMs but considers all large matrix positions instead of only the highest scoring one. This second algorithm (overviewed in Figure 6B) displays the best prediction rates when limiting the smaller matrix scanning to within 75 nt upstream and downstream of the larger matrix's position, with 2–4% higher sensitivity depending on the resolution (data not shown). Although similarly as effective as the 25A25 matrix within 10 nt, this approach displays a higher sensitivity when lowering the resolution to 100 nt (Figure 5C). Predictions are nonetheless higher than using individual matrices at any resolution. Including such an approach in a poly(A) prediction program is straightforward given its higher sensitivity.

In order to asses the selectivity of this approach, the highest scores obtained from annotated coding sequences (CDS) were compared to those of known splice-junction and poly(A) regions. The average highest score for poly(A) prediction in all 3789 *Leishmania infantum *CDS over 1500 nucleotides in length is 17.8 bits with a standard deviation of 7.8 bits. Using the same data, the average high score for SJ prediction is 704.1 units with a standard deviation of 401.1 units. The inherent properties of normal distribution statistics stipulate that over 95% of the high scores are within two standard deviations of the mean [35]. Thresholds corresponding to these values (e.g., 34 for poly(A) and 1506 for SJ prediction) were incorporated to the prediction algorithm, which then scanned all datasets. The resulting false-positive and true-positive detection rates are presented in Table 3. Integrating the scoring thresholds limits false-positive predictions to less than 5% while only slightly affecting specificity (predictions drop 5–6% for SJ and 1–2% for poly(A) predictions).

**Table 3:** Accuracy of mRNA extremity predictions.

	**Splice-Junction Prediction**		**Poly(A) Prediction**	
	**Exact**	**< 25 nt**	**< 10 nt**	**< 100 nt**
**Sensitivity**	48.6%	58.9%	25.2% (1.5)	45.4% (1.2)
Specificity*	96.4%		97.3%	

* Tested on all 3789 annotated coding sequences over 1500 nucleotides in length in *L. infantum*

True positive (sensitivity) and false positive (specificity) rates are depicted for the best splice-junction and poly(A) site prediction methods. Percentages represent the relative amount of hits that score above a statistical threshold derived from empirical data. The values in parentheses are the standard deviation for poly(A) site prediction sensitivities determined by 15 runs of ten fold cross-validation.

## Discussion

The 5' splice junction prediction methods disclosed in this work were conceived to estimate *trans*-splicing sites for all input sequences using a simple and effective metric. Since pyrimidines play an important role in *trans*-splicing, including such a parameter into the inter-AG splice prediction model was forthright and can be warranted by the subsequent increase in sensitivity. Although rather effective, the inter-AG metric's principal hoodwink resides in its synthetic nature, as the underlying biological process is difficult to conceive. The assessment of polypyrimidine tract length was not considered in this work as it has been shown that the inter-AG metric is more powerful [23]. Even if our splice junction prediction results are encouraging, some uncertainty subsists when testing on unconfirmed sequences. This may potentially be a consequence of the parasitic nature of trypanosomatids, which coerces these protozoa to alternate between different life-stages depending on their insect and mammalian host. An additional level of complexity may be essential to improve *in-silico *predictions in view of the fact that *trans*-splicing of certain transcripts is developmentally regulated in trypanosomes [36,37].

When compared to previously published *trans*-splicing prediction rates [23], the models we propose here appear to be just as effective at predicting known *trans*-splicing sites when tested on the same search space (Table 1). Their accuracy remains significant even when increasing the query sequence size (1.75× increase in search space at the cost of 0.9× accuracy). The augmented search space is in order to ensure that the full inter-AG fragments upstream of putative splice sites are considered. Overlapping into the downstream coding sequence is vindicated by erroneous genome annotations; it is not uncommon that the furthest in-frame ATG is selected as a start codon. Also, our scoring function rates all inter-AG fragments, unlike the previously proposed study that selects high-scoring fragments based upon their dinucleotide composition [23]. As shown in Table 3, a scoring threshold can be implemented to ensure that few false-positives are unsuitably identified as splice-junctions at the cost of slightly lower specificity. However, a threshold will necessarily neglect certain sequences, which may be objectionable when dealing with few or essential queries. Since our method is more dependent on correct annotations, it is conceivable that coupling it to linear discriminant analysis would generate even better predictions at the cost of higher complexity.

Predicting poly(A) sites with PSSM's have previously been shown to successfully predict poly(A) sites in humans [38]. Capturing the global nucleotide composition surrounding known poly(A) sites and utilizing it as a comparative predictor has also proven to be a successful prediction procedure in *Leishmania*. Albeit the public EST data appears to be of questionable quality, stringent screening has permitted to reveal specific polyadenylated sequence traits. Given the nature of the sequence data, smaller mRNA transcripts may be favoured and this should be considered when analyzing results. Nonetheless, PSSM scanning is more than 10 times more effective at identifying poly(A) sites than the distance-only approach when precision is fundamental (Figures 2A and 4). This result can be interpreted as evidence that distance is not as powerful for targeting poly(A) sites in *Leishmania *than in trypanosomes.

For *Leishmania*, precision may not be essential when predicting 3'UTR extremities given that several mappings display heterogeneous poly(A) positions [15]. This observation motivates the use of an error margin, which is interpreted as lowering the resolution of sensitivity testing in this work. Allowing correct predictions to be within a certain range of the mapped position emulates the identification of a polyadenylation region. We also tested a window scanning approach, where the cumulative bit-scores for a given range were averaged over the size of the window instead of considering each position independently. Such an approach yielded weaker overall predictions than the position-specific approach (data not shown), perhaps because the extent of polyadenylation regions varies among different transcripts.

The best 3'UTR predictions emanate from the grouping of distance limitation and scanning with dual PSSMs. Combining both metrics proved to be more effective than either one individually (Figures 2, 4, and 5), a result that hints at the importance of each factor when predicting poly-A sites in *Leishmania*. For restraining PSSM scanning, we tested various distances instead of using a specific confidence interval since spacer sequences display somewhat of a bias towards longer fragments. Although the data is partially derived from estimations, such a shift in the distribution supports the notion that polyadenylation does not occur randomly in *Leishmania*. Poly(A) sites further away from the splice junction may be an effect of distant polypyrimidine tracts, a situation that has already been observed in trypanosomes [20]. One must also consider that the longer non-coding regions in *Leishmania *may contain non-annotated genes or provide alternative stage-specific polyadenylation sites, which could explain the longer spacer sequences. These are considerations that motivated the exclusion of intergenic sequences longer than 5000 nucleotides for sensitivity testing.

To our knowledge, no other method can predict poly(A) sites as effectively in *Leishmania spp*. as the one described in this work. Even enforcing a highly-selective threshold only faintly affects this method's specificity (Table 3). The rather unusual and non-specific nature of kinetoplastid polyadenylation is a line of reasoning to substantiate low computational prediction rates. Although over-represented A-rich hexamer motifs are found (Additional File 2), these are not however present in all the genomic poly(A) sites, which suggests that they may not play a central role in driving polyadenylation in *Leishmania*. In addition, the genomic alignment of polyadenylated EST mappings cannot be used to mark out a precise consensus sequence, as it is impossible to distinguish the exact cleavage site among multiple consecutive adenosines on the unprocessed transcript. The heterogeneity of poly(A) sites in *Leishmania *mRNA transcripts is extra incentive for using PSSMs that embody a global trend in nucleotide composition. Furthermore, neglecting secondary structure and stage-specificity are additional factors that make it difficult to conceive obtaining higher prediction accuracies at this point.

Notwithstanding the possibility that no consensus motif drives polyadenylation in kinetoplastids, there is evidence for a biological model based on sequence context. The low sensitivity obtained from a poly(A) prediction algorithm based on spacing metrics alone is an evidence for a more dynamic biological model. Also, the correlation between certain regions of the genomic alignment and their respective prediction rates is most interesting, as best illustrated by the sensitivity surface plots (Figure 2). The data is presented in order to asses the innate characteristics that have an impact on poly(A) targeting.

Two main common sequence features appear to directly influence the prediction sensitivities. Firstly, the adenosine-rich region within close range to the mapped poly(A) site. Secondly, the pyrimidine-rich region 300 to 600 positions downstream. The latter, which represents the polypyrimidine tracts known to be crucial for *trans*-splicing, generates the best predictions when loosening the accuracy and bounding the search space. In turn, the A-rich region is responsible for the best predictions when precision is fundamental. Interestingly, the affluence of polypyrimidines (most notably thymines) in the -50 to -25 region (Figure 1) may play a role in 3'UTR cleavage since its exclusion from scanning matrices reduces the sensitivity at close range (Figure 2). The matrix encoding the sequence information of zero upstream bases and 25 downstream (0A25) is somewhat futile at predicting poly(A) sites, a rather surprising observation seeing as the adenosine concentration is comparable. Upon closer inspection, it is apparent that adenosine-rich regions are not a fundamental marker because many sequences do not contain profuse adenosine residues at their poly(A) site.

PSSMs can be regarded as a simplistic representation of the interaction between an enzymatic complex and a strand of nucleic acids. The highest scoring position corresponds to a region that is most similar to the consensus of all poly(A) sites, which relates to a high affinity region for the polyadenylation complex. In this perspective and based on our results, it is enticing to contemplate a generic biological model where adenosine richness (possibly contrasted by a pyrimidine-rich upstream region) helps to direct the polyadenylation of specific positions downstream of polypyrimidine tracts in unprocessed mRNA transcripts. Deletion studies directed at these features followed by mapping the modified transcript's poly(A) site could shed additional light into the biological process. Moreover, *in-vitro *UV cross-linking could help identifying novel ribonucleoproteins (RNPs) that might interact with the *trans*-splicing/polyadenylation complexes.

The computational tools we describe in this work have been implemented in a small JAVA program named PRED-A-TERM (PREDicting poly(A) sites and TERMinal splice junctions) that can be downloaded from Additional File 4. It emits poly(A) and *trans*-splicing predictions from intergenic sequence input with partial coding sequence overlap and allows end-users the possibility to select various prediction parameters. The program is tuned for *L. infantum *but is suitable for other *Leishmania *species. Although trypanosomes have shorter average intergenic regions than *Leishmania*, both share similar *trans*-splicing machinery [39,40]. Scanning *Trypanosoma *IRs will however, require additional sequence analysis and subsequent tuning of the model.

## Conclusion

We present a simplified 5'UTR prediction function that can predict more than 65% of known *trans*-splicing sites within 25 nucleotides. This approach performs as good as previously published methods but it significantly reduces computational cost. We also present a novel 3'UTR prediction method for the trypanosomatid *Leishmania *that compares query sequences to known polyadenylated sequences using position specific scanning matrices. Such an approach is capable of predicting almost 50% of known poly(A) sites within 100 nucleotides, thus doubling the accuracy of the previous distance based approach. The final algorithm implemented in PRED-A-TERM is summarized in Figure 6.

By increasing the precision of large-scale transcriptome predictions in trypanosomatids, the prospective identification of novel regulatory non-coding RNA structures is now within reach. The relatively recent fervour for investigating regulatory functions of non-coding RNA has propelled the emergence of multiple structural RNA detection algorithms [41,42]. These modern computational methods combined with biological validation could facilitate the discovery of innovative targets for therapeutic treatments.

## Methods

### 5' Splice junction prediction

After aligning the EST data to the genome, we extracted 500 nucleotides upstream of the coding sequence associated to the EST and the first 200 nucleotides downstream of the annotated start codon. *Trans*-splicing predictions are based upon the most recently published method [23]. Sequences are fragmented at every occurrence of "AG" and each fragment's size is calculated. In the simplest scoring scheme, the longest inter-AG fragment is retained and the sequence's final position is considered as a splice junction candidate. Linear and polynomial pyrimidine bias models calculate the relative pyrimidine concentration of inter-AG fragments and modify each fragment's score proportionately using the following functions:

***L ***= λ + 150•λ•δ

***P ***= λ + 150•λ•δ^3^

where ***L ***and ***P ***are the linear and polynomial model scores respectively, λ is the inter-AG fragment length, and δ corresponds to the difference between the pyrimidine concentration of the inter-AG fragment and the average intergenic concentration (55%). In both cases, the last position of the highest scoring inter-AG fragment is retained as the putative splice junction. Optimal coefficients were determined by trial and error testing. Sensitivity testing was performed on the same 214 EST sequences from *Leishmania major *as reported in that article.

### Data collection

*Leishmania *sequences for the poly(A) analysis were downloaded from GenBank's expressed sequence tag (EST) public database [43]. Data were filtered to retain sequences having at least 12 adenine (A) or thymine (T) residues at their 3' or 5' end, respectively. Poly-T sequences were subsequently reverse-complemented. *Leishmania infantum *sequences were aligned to the genome (version 3 downloaded from [44]) using BLAST with low-complexity filtering disabled [45]. Hits over 100 nucleotides long that displayed over 95% sequence identity were retained. We define an EST sequence as being polyadenylated if it satisfies the following criteria: (i) The last position of the best BLAST hit must immediately precede the poly(A) stretch. (ii) There should be no more than 9 "A" residues out of the next 12 genomic nucleotides following the BLAST hit. (iii) The last alignment match must not be a "N" in the genomic or EST sequence. The polyadenylation site is defined as the last non-"A" residue shared between the EST extremity and the genomic sequence. The full list of polyadenylated EST accession numbers can be viewed in Additional File 1. All filtering steps were achieved using *ad-hoc *JAVA scripts.

### Building poly(A) scanning matrices

The genomic sequences of the polyadenylated ESTs were aligned and anchored at the mapped poly(A) site, as previously defined. From this alignment, we calculated the specific nucleotide composition for each position relative to the poly(A) site. The resulting nucleotide frequencies were divided by their corresponding average genomic frequency (A = 20.1%, T = 20.2%, C = 29.7%, G = 30.0%) to create an odds matrix. The final position specific scoring matrix (PSSM) was obtained by log-transforming the odds matrix to generate bit scores for each matrix entry.

### Poly(A) prediction using scanning matrices

The genomic intergenic regions (IRs) associated to the retained ESTs were extracted and extended 600 bases past the stop and start codons of the most recent *L. infantum *genome annotation (version 3). Only IRs inferior or equal to 5000 bases in length were retained. IRs of interest were scanned with PSSM sizes ranging from 1 to 300 upstream and 1 to 600 downstream of the poly(A) location. When scanning the entire IR, query sequences are scanned such that the position corresponding to the anchored poly(A) site in the PSSM is aligned to the first non-coding position downstream of the stop codon; at this point, the upstream matrix positions overlap the ORF. A cumulative bit-score is emitted for each given position and this step is repeated for every position of the intergenic sequence (the positions downstream of the matrix's poly(A) position may overlap the ORF when scanning the last positions). The positions with the highest scores are retained as putative polyadenylation sites. When predicting a polyadenylation region instead of a single position, the cumulative individual bit-scores are averaged over the length of the region scanned. The optimal prediction algorithm is summarized in Figure 6.

### Ten-fold cross-validation sensitivity testing

The prediction accuracies presented in this work arise from cross-validation sensitivity testing, where the polyadenylated EST data are divided into 10 subsets. Nine of those are used as a training set (in this case, to build a PSSM) which are subsequently tested on the left-over subset. This step is repeated for all subsets and the results are averaged to obtain the mean sensitivity. The average and standard deviation of 15 runs of cross-validation were performed for PSSM scanning and 30 runs for distance-only predictions. All testing was performed using *ad-hoc *JAVA scripts.

## Authors' contributions

MS conceived of the study, performed all computational analyses, and drafted the manuscript. MB and BP contributed to the conception and coordination of the study and helped draft the manuscript. All authors read and approved the final manuscript.

## Supplementary Material

Additional file 1 **Polyadenylated ESTs**. PDF document of all 218 polyadenylated EST accession IDs used to build scanning matrices in this work. Click here for file

Additional file 2 **Over-represented hexamers**. A Microsoft Word document containing the 10 highest scoring hexamers identified with YMF and FindExplanator programs. An alignment of 223 sequences was used to compare regions encompassing the [-125; +125] of genomic poly(A) sites to the [-800; -126] and [+126; +800] regions. Click here for file

Additional file 3 **PSSM scanning results for different matrix sizes and scanning distances**. A Microsoft Excel spreadsheet containing all sensitivity results for single-matrix scanning approaches. Click here for file

Additional file 4 **PRED-A-TERM program**. The prediction algorithm described in this manuscript has been implemented into a JAVA program which can be used to scan query sequences using any operating system. Once extracted, detailed usage instructions can be viewed in the README.txt file. Click here for file
