# Supplementary material for: Improving the prediction of mRNA extremities in the parasitic protozoan Leishmania
Source: BMC Bioinformatics. 2008 Mar 20;9:158. doi: 10.1186/1471-2105-9-158 (PMC2335281; doi:10.1186/1471-2105-9-158)
Supplement: Additional file 1 — Polyadenylated ESTs. PDF document of all 218 polyadenylated EST accession IDs used to build scanning matrices in this work. [file 1471-2105-9-158-S1.pdf]

**Additional File 1 – GenBank accession Ids of polyadenylated ESTs for *Leishmania infantum***
See Methods for more information on search parameters.

CV670622
CV670278
CV670124
CV670078
CV670050
CV670037
CV670031
CV670015
CV670011
CV669673
CV669580
CV669553
CV665459
CV669548
CV669103
CV668879
CV668764
CV668103
CV667818
CV667706
CV667665
CV667646
CV667639
CV667625
CV667619
CV667005
CV666662
CV666468
CV662176
CV667598
CV667575
CV667542
CV667358
CV667347
CV667338
CV667325
CV667210
CV667167
CV667149
CV667147
CV667104
CV667102
CV667085
CV666990
CV666905
CV666863
CV666798
CV666794
CV666789
CV666769
CV666760
CV666735
CV666724
CV666719
CV666609
CV666605
CV664280
CV670675
CV670639
CV670623
CV670467
CV670463
CV670444
CV670436
CV670417
CV670284
CV670279
CV670119
CV670097
CV669957
CV669949
CV669922
CV669890
CV669846
CV669835
CV669821
CV669819
CV669799
CV669790
CV669752
CV669741
CV669706
CV669691
CV669653
CV669592
CV669583
CV669563
CV669476
CV669453
CV669387
CV669248
CV669211
CV669091
CV668957
CV668840
CV668518
CV668486
CV668435
CV668434
CV668356
CV668213
CV668181
CV668178
CV668130
CV668096
CV668014
CV667999
CV667777
CV667565
CV667515
CV667398
CV667385
CV667347
CV667232
CV666826
CV666690
CV666586
CV666576
CV666568
CV666518
CV666500
CV666454
CV666429
CV666427
CV666389
CV666323
CV666258
CV666121
CV666101
CV666093
CV666087
CV666084
CV666073
CV666071
CV666056
CV666038
CV665948
CV665911
CV665909
CV665901
CV665859
CV665846
CV665815
CV665766
CV665702
CV665682
CV665566
CV665540
CV665362
CV665328
CV665288
CV665232
CV665225
CV665041
CV665020
CV664992
CV664948
CV664863
CV664795
CV664644
CV664627
CV664599
CV664591
CV664588
CV664585
CV664554
CV664463
CV664400
CV664287
CV664282
CV664268
CV664255
CV664242
CV664154
CV664102
CV664094
CV664028
CV664006
CV663984
CV663977
CV663968
CV663966
CV663941
CV663871
CV663850
CV663778
CV663715
CV663605
CV663591
CV663579
CV663564
CV663464
CV663132
CV662998
CV662943
CV662751
CV662543
CV662518
CV662516
CV662488
CV662290
CV662231
CV662152
CV662127
CV661979
CV661923
CV661857
CV661842
CV661832
CV661828
CV661695
CV661552
CV661459
CV661168
CV660981
CV660962
CV660927
AJ276158
