# Supplementary material for: Improving the prediction of mRNA extremities in the parasitic protozoan Leishmania
Source: BMC Bioinformatics. 2008 Mar 20;9:158. doi: 10.1186/1471-2105-9-158 (PMC2335281; doi:10.1186/1471-2105-9-158)
Supplement: Additional file 2 — Over-represented hexamers. A Microsoft Word document containing the 10 highest scoring hexamers identified with YMF and FindExplanator programs. An alignment of 223 sequences was used to compare regions encompassing the [-125; +125] of genomic poly(A) sites to the [-800; -126] and [+126; +800] regions. [file 1471-2105-9-158-S2.doc]

**Additional File 2**

List of over-represented motifs contained in the [-125; +125] region flanking genomic poly(A) sites when compared to the [-800; -126] and [+126; +800] regions, as identified by YMF + FindExplanator (see manuscript for references).

| **Hexamer** | **Z-score** | **Hexamer** | **Z-score** |
| --- | --- | --- | --- |
| AAACAA | 13.0 | GAGAGA | 7.3 |
| ACACAC | 12.7 | AAAGAA | 7.4 |
| ATACAT | 9.2 | AAGCAA | 6.7 |
| CACGCA | 8.3 | CCCTCC | 6.6 |
| AACCAA | 7.4 | GCTAGC | 6.2 |
